# Consulting Obese and Overweight Patients for Nutrition and Physical Activity in Primary Healthcare in Poland

**DOI:** 10.3390/ijerph19137694

**Published:** 2022-06-23

**Authors:** Małgorzata Znyk, Radosław Zajdel, Dorota Kaleta

**Affiliations:** 1 Department of Hygiene and Epidemiology, Medical University of Lodz, Żeligowskiego 7/9, 90-752 Łódź, Poland; dorota.kaleta@umed.lodz.pl; 2 Department of Computer Science in Economics, University of Lodz, POW 3/5, 90-255 Łódź, Poland; radoslaw.zajdel@uni.lodz.pl

**Keywords:** GP doctor, Poland, nutrition, physical activity, overweight, obesity, counseling

## Abstract

The aim of this study was to evaluate the dietary and physical activity counseling provided to adults by family doctors. Predictors of counseling in primary healthcare were identified. A cross-sectional study was conducted from January 2020 to December 2021 among 896 adult primary care patients in the city of Łódź [Lodz], Poland. Almost 36% of the respondents were advised to change their eating habits, and 39.6% were advised to increase their physical activity. In a multivariate logistic regression analysis, people in poor health with chronic diseases related to overweight and obesity and with two, three or more chronic diseases, respectively, received advice on eating habits from their GP twice and three times more often than people in good health with no chronic conditions (OR = 1.81; *p* < 0.05 and OR = 1.63; *p* < 0.05; OR = 3.03; *p* < 0.001). People in the age groups 30–39 years and 40–49 years (OR = 1.71; *p* < 0.05 and OR = 1.58; *p* < 0.05), widowed (OR = 2.94; *p* < 0.05), with two, three or more chronic diseases (OR = 1.92; *p* < 0.01 and OR = 3.89; *p* < 0.001), and subjectively assessing overweight and obesity (OR = 1.61; *p* < 0.01) had a better chance of receiving advice on physical activity. The study found a higher proportion of advice on diet and physical activity provided to overweight and obese patients by primary care physicians than in other studies; however, still not all receive the necessary counseling. GPs should advise all patients not to become overweight and obese, not only those already affected by the problem.

## 1. Introduction

Overweight and obesity are one of the main risk factors for chronic diseases, including cardiovascular diseases, diabetes, certain cancers, disorders of the musculoskeletal system, and disability [1,2,3]. Obesity is a serious public health problem with increasing social, health, and economic costs [4,5].

Worldwide, the number of people who are overweight and obese has grown in all age groups and is expected to rise even further over the next decade. According to the World Health Organization (WHO), in 2020, the number of adults with overweight was 1.9 billion, and those with obesity 0.6 million [6]. In 2019, 52.7% of the adult population in the European Union were overweight, and among them, 17% were people suffering from obesity [7]. The percentage of overweight adults varies across countries, with the highest proportions in Croatia and Malta, where 65% of adults were considered overweight in 2019. In Poland, this percentage is 58.1%, while the aforementioned European average is 52.7% [7,8]. It is estimated that in total 50–70% of the adult Polish population are overweight and obese, including over 25% of obesity cases. In 2020, 54% of Polish people were overweight (46% of women and 64% of men) [9]. The high body mass index (BMI ≥ 25) in Poland accounts for 14.2% of deaths (15.3% of women and 13.1% of men) [9]. These data indicate that overweight and obesity are significant problems in the Polish population; therefore, it is necessary to implement strong measures to prevent them. It means that actions should be taken both at the individual and population levels [10].

Doctors play a major role in the diagnosis and treatment of overweight and obesity [5]. The basic method of therapy in these conditions is still dietary treatment combined with increased physical activity [11]. The beneficial effects of combined dietary interventions and physical activity have been observed in numerous studies on obese patients in primary care [12,13,14]. This approach has been shown to be effective for reducing obesity and cardiovascular risk factors.

Improving obesity treatment in primary care settings may reduce the incidence of comorbidities and enhance patients’ quality of life [4]. It can also prevent premature aging and extend patients’ lives.

In Poland, primary healthcare is the first point of contact with a doctor. Primary healthcare doctors are mainly general practitioners who provide comprehensive care for patients. This type of healthcare service is available to everyone who is insured by the National Health Fund [15].

It should be remembered that patients choosing a specific family doctor remain under his/her care for many years. A bond is created between the doctor and his/her patient and the relationship should be built on trust [16]. However, if a patient is mistreated by his/her GP, there is a risk of chronic distrust.

Counseling by a primary care physician may change pro-health behaviors related to weight, such as diet and physical activity in adult patients [17,18,19,20]. However, the percentage of advice on healthy behavior given by family doctors to overweight people is still low. Lifestyle-related issues were raised in every third person provided with primary healthcare [21]. The low percentage of discussions about weight control suggests that GPs may not be using their full potential in the fight against obesity [22].

Primary care guidelines for overweight and obese adults recommend routine measurements of body mass index [23,24]. According to the guidelines recommended in Poland, adult screening tests (BMI calculation) for overweight and obesity should be carried out annually. An integral part of the physical examination performed by a GP should be measurements of height, weight, and waist circumference, as well as BMI calculations. After making the diagnosis, the family doctor should talk to the patient about the benefits of weight loss and the risk of obesity complications [5]. Treating obesity during the COVID-19 pandemic became even more important than before, since some people tried to compensate for negative emotions with food. The algorithm for the procedure applied in a doctor’s office should consider the general health of the patient [25].

Education about a healthy diet and physical activity, especially by a family doctor, can change the patient’s individual health behavior, and thus reduce the occurrence of metabolic diseases. Little is known about diet and physical activity counseling in primary healthcare in Poland, and this knowledge is essential to public health. The assessment of health-related counseling can identify current practices, indicate potential areas of intervention, influence relevant preventive measures, and contribute to reducing overweight and obesity in the population. Preventive measures in Poland are action-based. The National Health Fund does not provide any data specifying the scope of dietary and physical activity counseling offered, and it may affect patient satisfaction as well as assessments of the quality of the provided advice.

The aim of this study was to evaluate dietary and physical activity counseling provided by primary care physicians to adults.

## 2. Materials and Methods

### 2.1. Study Design and Population

A cross-sectional study was conducted from January 2020 to December 2021 among adult primary care patients in the city of Lodz, Poland.

Lodz is a Polish city with the worst health indicators among its inhabitants. Generally, in Poland, the inhabitants of the largest cities live the longest, with the exception of the city of Lodz, where the inhabitants live even shorter than those residing in small towns [9]. In the past, the WOBASZ I (2003–2006) and WOBASZ II (2013–2014) Multicenter National Population Health Examination Surveys were conducted in Poland. The surveys also covered the inhabitants of Lodz [26].

The number of general practitioners in the Lodz province is 433 (as of 31 December 2019) [27]. According to data received from the National Health Fund, in 2020 and 2021, there were 211 primary healthcare entities in the city of Lodz. Both the number of entities and the number of primary care physicians in Lodz are low, as is the case in the whole territory of Poland. As compared to other countries, Poland has the lowest share of GPs (which is only 9% of all specialists), except for Greece (6%) [28].

From the list of 211 primary health care facilities, every fifth clinic was selected randomly. The selection process was conducted to guarantee geographical representation. Thirty-four primary healthcare facilities agreed to conduct the study among their patients. From the randomly selected clinics, eight refused to participate. 

The required sample size was calculated for two-sided tests at a significance level of 0.05 and power for selected alternative hypothesis equal to 0.8. For OR = 1.5 and a hypothetical proportion of controls with exposure equal to 30%, about 855 participants are required. 

Adults over 18 years of age who consulted a doctor in primary care and agreed to participate in the study were included in the study. Individuals under the age of 18 years and adults who did not give their consent were excluded from the study. The participation rate was 80%.

On Monday and Wednesday in the morning and on Tuesday and Friday in the afternoon, every fifth patient leaving the doctor’s office was randomly selected and asked to enter the study. In case he/she refused consent another sixth patient was selected. The study included 896 patients who gave their written consent to participate in the study, whereas 221 refused to participate. In each facility that agreed to examine patients, 26–27 individuals were examined. At the same time, a questionnaire survey was conducted among family doctors. The results will be presented in the following articles. The study was approved by the Bioethics Committee of the Medical University of Lodz on 18 September 2018 (RNN/315/18/KE).

### 2.2. Study Variables

The principal researcher conducted data collection. The research tool was an anonymous paper-based questionnaire including mainly closed questions. It was based on standardized questions that had been used in other studies [29,30].

Face-to-face interviews were conducted. The survey included questions regarding sociodemographic features and information on appointments with primary care physicians and nurses, as well as characteristics of lifestyle factors.

The following socio-demographic variables were selected for the study: gender, age, education, marital status, and employment status. The article covers two sections out of seven included in the questionnaire, i.e., information about appointments with a primary healthcare doctor and information on the role of a physician as a provider of healthy lifestyle.

Information on the family doctor talking to the patient about eating habits and physical activity was obtained based on the following survey questions: “Has the family doctor ever talked to you about your eating habits or diet?”, and “Has the family doctor ever talked to you about physical activity and exercise?”. Additionally, the respondents were asked whether the topic was initiated by them or a family doctor.

Information on receiving advice from a family doctor was obtained based on the following questions: “How often does your doctor advise you on a healthy diet/proper nutrition”, and “How often does your doctor advise you to be physically active?”.

People who have never been counseled on eating habits, diet, or physical activity are respondents who answered “never” to the above questions, and those who answered “sometimes”, “often”, or “always “, were classified as patients who received counseling in the above-mentioned issues.

The answer “sometimes” concerned less than 50% of advice during all medical appointments in primary care and “often” 50% or more of advice during all medical appointments in primary care, whereas the answer “always” related to advice at each visit to a primary care physician.

The questionnaire also included questions about the frequency of measuring body weight on a scale, height, and waist circumference, and calculation of their body mass index (BMI) by a family doctor. The possible options were: (1) at each routine visit, (2) annually, (3) if clinically indicated, and (4) never.

The patients were asked to provide information on their height (m) and weight (kg).

Based on the height (m) and weight (kg), the BMI (kg/m^2^) for each respondent was calculated according to the formula: weight (kg) divided by height squared (m^2^) [31]. The study subjects were divided into three groups according to BMI: <25 kg/m^2^ (normal), ≥25–<30 kg/m^2^ (overweight), and ≥30 kg/m^2^ (obese), in compliance with the WHO recommendations. The following categories were applied: normal BMI (18.5–24.9 kg/m^2^), overweight (25.0–29.9 kg/m^2^), obesity class I (light) (30.0–34.9 kg/m^2^), and obesity classes II and III (severe) (≥35.0 kg/m^2^).

The respondents were asked about chronic diseases related to overweight and obesity, such as coronary artery disease, hypertension, type II diabetes, chronic obstructive pulmonary disease or asthma, and others, treated by a family doctor. Based on the answers given, they were divided into four groups: no disease, one, two, and three or more diseases.

The study participants were also asked about their subjective assessment of being overweight and obese. Table 1 presents the characteristics of the studied population.

### 2.3. Statistical Analysis

Descriptive statistics and the distribution of the studied variables were carried out. The data were presented as numbers and percentage rates. Categorical variables as a percentage were compared using the chi-square test. Single-variable and multivariate logistic regression analyses were performed to obtain ORs (odds ratios) and 95% confidence intervals (CIs) of each indicator for diet and exercise counseling. Variables with *p* values of 0.1 or less from the univariate analysis were included in the multivariate model. A *p*-value of less than 0.05 was considered statistically significant. The analyses were carried out using STATISTICA version 13.3. Missing values were removed in pairs.

## 3. Results

### 3.1. Characteristics of the Studied Population

Among the respondents, 25.8% were men and 74.2% were women (Table 1). Most of the study participants had secondary (56.9%) and higher (31.9%) education levels. The most numerous groups of respondents were people aged <30 years (28.6%) and the group of those aged 40–49 years (24.0%). Five percent of the respondents were unemployed, and professional activity was reported by 61.6%. Out of 896 adult primary care patients in the city of Lodz, 25.4% were overweight and 19.5% were obese. In addition, 23.7% of the respondents admitted that they suffered from chronic diseases, i.e., cardiovascular diseases, and type II diabetes (Table 1). In a subjective assessment, 32.8% of the respondents stated that they were overweight or obese. The response rate was high (80%) as compared to other surveys conducted in Poland. There was no lack of data in the responses of the subjects included in the analysis.

### 3.2. Advice on Nutrition and Exercise

A total of 36% of respondents consulted their primary care physicians about their eating habits or diet, and 39.6% consulted them about physical exercises. Obese respondents were more likely to talk to their family doctor about nutrition and physical activity as compared to overweight people (Table 2). In addition, 60% of obese and 39.2% of overweight patients spoke to their doctors about their eating habits or diet. Among the study group, 60% of obese and 41% of overweight subjects spoke about physical activity and exercise.

Approximately 71% of the discussions on eating habits and diet, as well as physical activity and exercise, were initiated by the family doctor. The family doctor opened a conversation about eating habits or diets more often in obese and overweight people (77.1% and 75.3%, respectively). Similarly, a conversation about physical activity and exercise was more often undertaken by a physician for obese and overweight people (76.2% and 66.7%). Overweight and obese respondents who talked to the doctor most often indicated that the family doctor sometimes advised them on diet, proper nutrition, and physical activity.

Among the respondents, 29.1% of obese and 23.4% of overweight patients indicated that their doctors sometimes advised them on a healthy diet or proper nutrition, while 27.4% and 23.8% of the respondents received advice on physical activity. In addition, 30.8% of obese and 22.0% of overweight patients sometimes received general advice on changing their diet, exercise, or weight loss; 29.7% of obese and 20.7% of overweight patients were given detailed advice on diets/nutrition; and 29.1% and 20.7% were given advice on physical activity. Sometimes the family doctor gave detailed advice on weight control (in 32.0% of obese and 18.1% of overweight patients).

The association between personal characteristics (age, gender, education, marital status, professional status, place of residence, and financial situation) and counseling was examined using logistic regression analysis. The odds ratio (OR) and a 95% confidence interval (Cl) were used to measure the strength of the association. The results of the univariate and multivariate logistic regression analyses for GP counseling with socio-demographic and health correlates are presented in Table 3.

In the univariate analysis, the variables such as a male gender, age 60+, and two chronic diseases were statistically significant (*p* < 0.001), and statistically insignificant in the multivariate logistic regression analysis.

In the multivariate logistic regression analysis, variables that were statistically significant in the univariate logistic regression analysis were considered.

In the multivariate logistic regression analysis, individuals in poor health with chronic diseases related to overweight and obesity and with two chronic diseases and three or more chronic diseases, respectively, received advice on eating habits twice and three times more often from their GP than people in good health with no chronic conditions (OR = 1.81; *p* < 0.05 and OR = 1.63; *p* < 0.05; OR = 3.03; *p* < 0.001). The widowed (OR = 2.43; *p* < 0.05) and those with subjectively assessed overweight and obesity (OR = 1.30; *p* < 0.05) had a better chance of getting advice on diets.

People in the age groups 30–39 years and 40–49 years (OR = 1.71; *p* < 0.05 and OR = 1.58; *p* < 0.05), widowed (OR = 2.94; *p* < 0.05), with two chronic diseases or three and more chronic diseases (OR = 1.92; *p* < 0.01 and OR = 3.89; *p* < 0.001), and subjectively assessed overweight and obesity (OR = 1.61; *p* < 0.01) had a better chance of getting advice on physical activity. Gender, education, marital status, professional situation, and diseases occurring in the family in the multivariate logistic regression analysis did not increase the chance of getting advice on diet or physical activity.

### 3.3. Body Weight Measurement

Among the respondents, 9.6% of individuals indicated that their body weight was measured using a scale by a GP once a year. Once a year, height was measured in 11.6% of the patients, and waist circumference was measured in 3.8% of the subjects. Forty two percent of the respondents indicated that body weight was measured in case of clinical indications, similarly to height and waist circumference (Table 4).

According to the respondents, the body mass index was calculated by a physician mainly in the case of clinical indications (25.3%). Moreover, 7.3% of the respondents reported BMI measurement once a year.

In the study group, 2.3% of the obese and 1.3% of the overweight subjects were prescribed medication for weight loss, whereas 1.1% of the obese and 0.4% of the overweight individuals were referred for obesity surgery.

**Table 2:** Percentage of primary care patients whose GP advised changing eating habits and physical activity.

Variable	Body Mass Index BMI									Total *n* = 896		
	<25 kg/m^2^ *n* = 494			≥25–<30 kg/m^2^ *n* = 227			≥30 kg/m^2^ *n* = 175					
	*n* (%)	95% Cl	*p*-Value	*n* (%)	95% Cl	*p*-Value	*n* (%)	95% Cl	*p*-Value	*n* (%)	95% Cl	*p*-Value
**Has your GP ever talked to you about your eating habits or diet?**												
Yes	128 (25.9)	(22.0–29.8)	0.2591	89 (39.2)	(32.9–45.6)	0.3920	105 (60.0)	(52.7–67.3)	0.6000	322 (36.0)	(32.8–39.1)	0.3594
No	366 (74.1)	(70.2–78.0)	0.7409	138 (60.8)	(54.4–67.1)	0.6079	70 (40.0)	(32.7–47.3)	0.4000	574 (64.0)	(60.9–67.2)	0.6406
**How did the interview take place?**												
on your initiative	45 (35.2)	(26.9–43.4)	0.0911	22 (24.7)	(15.8–33.7)	0.0969	24 (22.9)	(14.8–30.9)	0.1371	91 (28.3)	(23.3–33.2)	0.1015
on the doctor’s initiative	83 (64.8)	(56.6–73.1)	0.1680	67 (75.3)	(66.3–84.2)	0.2952	81 (77.1)	(69.1–85.2)	0.4628	231 (71.7)	(66.8–76.7)	0.2578
**Has a GP ever talked to you about physical activity and exercise?**												
Yes	157 (31.8)	(27.7–35.9)	0.3178	93 (41.0)	(34.6–47.4)	0.4097	105 (60.0)	(52.7–67.3)	0.6000	355 (39.6)	(36.4–42.8)	0.3962
No	337 (68.2)	(64.1–72.3)	0.6822	134 (59.0)	(52.6–65.4)	0.5903	70 (40.0)	(32.7–47.3)	0.4000	541 (60.4)	(57.2–63.6)	0.6038
**How was the topic broached?**												
on your initiative	47 (29.9)	(22.8–37.1)	0.0951	31 (33.3)	(23.8–42.9)	0.1366	25 (23.8)	(15.7–32.0)	0.1428	103 (29.0)	(24.3–33.7)	0.1149
on the doctor’s initiative	110 (70.1)	(62.9–77.2)	0.2227	62 (66.7)	(57.1–76.2)	0.2731	80 (76.2)	(68.0–84.3)	0.4571	252 (71.0)	(66.3–75.7)	0.2812
**How often does the doctor advise you on**												
**a healthy diet/proper nutrition?**												
Never	379 (76.8)	(73.0–80.4)	0.7672	148 (65.2)	(59.0–71.4)	0.6520	96 (54.9)	(47.5–62.2)	0.5486	623 (69.5)	(66.5–72.5)	0.6953
Sometimes	87 (17.6)	(14.3–21.0)	0.1761	53 (23.4)	(17.8–28.9)	0.2335	51 (29.1)	(22.4–35.9)	0.2914	191 (21.3)	(18.6–24.0)	0.2132
Often	19 (3.8)	(2.2–5.5)	0.0385 *	20 (8.8)	(5.1–12.5)	0.0881	24 (13.7)	(8.6–18.8)	0.1371	63 (7.1)	(5.4–8.7)	0.0703
Always	9 (1.8)	(0.6–3.0)	0.0182 *	6 (2.6)	(0.6–4.7)	0.0264 *	4 (2.3)	(0.1–4.5)	0.0228 *	19 (2.1)	(1.2–3.1)	0.0212 *
**How often does the doctor advise you on physical activity?**												
Never	382 (77.3)	(73.6–81.0)	0.7733	150 (66.1)	(17.8–28.9)	0.6608	101 (57.7)	(50.4–65.0)	0.5771	633 (70.6)	(67.7–73.6)	0.7065
Sometimes	76 (15.4)	(12.2–18.6)	0.1538	54 (23.8)	(18.2–29.3)	0.2379	48 (27.4)	(20.8–34.0)	0.2742	178 (19.9)	(17.3–22.5)	0.1987
Often	29 (5.9)	(3.8–7.9)	0.05870	18 (7.9)	(4.4–11.4)	0.0793	23 (13.2)	(8.1–18.1)	0.1314	70 (7.8)	(6.1–9.6)	0.0781
Always	7 (1.4)	(0.4–2.5)	0.0142 *	5 (2.2)	(0.3–4.1)	0.0203 *	3 (1.7)	(−0.2–3.6)	0.0171 *	15 (1.7)	(0.8–2.5)	0.0167 *
**How often does a family doctor** **give you general advice on changing your diet, exercise, or weight loss?**												
Never	431 (87.2)	(84.3–90.2)	0.8725	163 (71.8)	(66.0–77.7)	0.7181	102 (58.3)	(51.0–65.6)	0.5828	696 (77.7)	(75.0–80.4)	0.7768
Sometimes	45 (9.1)	(6.6–11.6)	0.9109	50 (22.0)	(16.6–27.4)	0.2203	54 (30.8)	(24.0–37.7)	0.3086	149 (16.6)	(14.2–19.1)	0.1663
Often	17 (3.5)	(1.8–5.0)	0.0344 *	12 (5.3)	(2.4–8.2)	0.0529	15 (8.6)	(4.4–12.7)	0.0857	44 (4.9)	(3.5–6.3)	0.0491 *
Always	1 (0.2)	(−0.2–0.6)	0.0020 **	2 (0.9)	(−0.3–2.1)	0.0088	4 (2.3)	(0.1–4.5)	0.0228 *	7 (0.8)	(0.2–1.4)	0.0078
**How often does a family doctor give you detailed advice on diets/nutrition?**												
Never	402 (81.4)	(77.9–84.8)	0.8138	160 (70.4)	(64.6–76.4)	0.7048	103 (58.9)	(51.6–66.1)	0.5885	665 (74.2)	(71.4–77.1)	0.7422
Sometimes	69 (13.9)	(10.9–17.0)	0.1397	47 (20.7)	(15.4–26.0)	0.2070	52 (29.7)	(22.9–36.5)	0.2971	168 (18.7)	(16.2–21.3)	0.1875
Often	21 (4.3)	(2.5–6.0)	0.0425*	19 (8.4)	(4.8–12.0)	0.0837	16 (9.1)	(4.9–13.4)	0.0914	56 (6.3)	(4.7–7.8)	0.0625
Always	2 (0.4)	(−0.2–1.0)	0.0040 **	1 (0.5)	(−0.4–1.3)	0.0044 **	4 (2.3)	(0.1–4.5)	0.0228 *	7 (0.8)	(0.2–1.4)	0.0078 **
**How often does a family doctor give you detailed advice on physical activity?**												
Never	416 (84.2)	(81.0–87.4)	0.8421	170 (74.9)	(69.2–80.5)	0.7489	106 (60.6)	(53.3–67.8)	0.6057	692 (77.2)	(74.5–80.1)	0.7723
Sometimes	56 (11.3)	(8.5–14.1)	0.1134	47 (20.7)	(15.4–26.0)	0.2070	51 (29.1)	(22.4–35.9)	0.2914	154 (17.2)	(14.7–19.7)	0.1719
Often	20 (4.1)	(2.3–5.8)	0.0448 *	8 (3.5)	(1.1–5.9)	0.0352 *	14 (8.0)	(4.0–12.0)	0.0800	42 (4.7)	(3.3–6.1)	0.0469 *
Always	2 (0.4)	(−0.2–1.0)	0.0040 **	2 (0.9)	(−0.3–2.1)	0.0088 **	4 (2.3)	(0.1–4.5)	0.0228 *	8 (0.9)	(0.3–1.5)	0.0089 **
**How often a family doctor gives you detailed advice on weight control?**												
Never	441 (89.3)	(86.5–92.0)	0.8927	171 (75.3)	(69.7–80.9)	0.7533	100 (57.1)	(49.8–64.5)	0.5714	712 (79.5)	(76.8–82.1)	0.7946
Sometimes	37 (7.5)	(5.2–9.8)	0.0749	41 (18.1)	(13.1–23.1)	0.1806	56 (32.0)	(25.1–38.9)	0.3200	134 (15.0)	(12.6–17.3)	0.1495
Often	12 (2.4)	(1.1–3.8)	0.0243 *	12 (5.3)	(2.4–8.2)	0.0528	14 (8.0)	(4.0–12.0)	0.0800	38 (4.2)	(2.9–5.6)	0.0424 *
Always	4 (0.8)	(0.01–1.6)	0.0081 **	3 (1.3)	(−0.2–2.8)	0.0132 *	5 (2.9)	(0.4–5.3)	0.0286 *	12 (1.3)	(0.6–2.1)	0.0133 *

* *p* < 0.05, ** *p* < 0.01.

**Table 3:** The odds ratio of receiving advice from a primary care physician according to the analyzed variables.

Variables	Advice on Changing Your Eating Habits *n* = 481					Advice for Increasing Physical Activity *n* = 480				
		Unadjusted Model		Adjusted Model			Unadjusted Model		Adjusted Model	
	*n* (%)	OR	95% Cl	OR	95% Cl	*n* (%)	OR	95% Cl	OR	95% Cl
**Gender**										
Female	332 (49.9)	1.00	Ref.	1.00	Ref.	329 (49.5)	1.00	Ref.	1.00	Ref.
Male	149 (64.5)	1.82	(1.33–2.48) ***	1.30	(0.91–1.85)	151 (65.4)	1.93	(1.41–2.63) ***	1.36	(0.95–1.95)
**Age (years)**										
<30	111 (43.4)	1.00	Ref.	1.00	Ref.	104 (40.6)	1.00	Ref.	1.00	Ref.
30–39	56 (45.5)	1.09	(0.71–1.68)	1.18	(0.71–1.93)	59 (48.00)	1.34	(0.87–2.08)	1.71	(1.04–2.83) *
40–49	110 (51.2)	1.36	(0.95–1.97)	1.17	(0.78–1.78)	114 (53.0)	1.65	(1.14–2.38) **	1.58	(1.04–2.40) *
50–59	58 (55.2)	1.61	(1.02–2.55) *	1.04	(0.58–1.88)	58 (55.2)	1.80	(1.14–2.85) *	1.42	(0.78–2.59)
60+	146 (74.1)	3.74	(2.50–5.60) ***	1.31	(0.64–2.69)	145 (73.6)	4.08	(2.72–6.10) ***	1.56	(0.76–3.23)
**Education**										
Primary	18 (69.2)	2.52	(1.06–5.98) *	1.32	(0.48–3.62)	19 (73.1)	3.12	(1.27–7.67) *	1.55	(0.55–4.34)
Medium/Secondary	282 (55.3)	1.38	(1.03–1.85) *	1.10	(0.78–1.56)	289 (56.7)	1.50	(1.12–2.01) **	1.24	(0.88–1.75)
Post-secondary vocational	46 (62.2)	1.83	(1.09–3.11) *	1.52	(0.86–2.71)	39 (52.7)	1.28	(0.77–2.14)	1.02	(0.58–1.81)
Higher	135 (47.2)	1.00	Ref.	1.00	Ref.	133 (46.5)	1.00	Ref.	1.00	Ref.
**Marital status**										
Single	179 (45.4)	1.13	(0.65–1.97)	1.39	(0.74–2.63)	184 (46.7)	1.19	(0.68–2.07)	1.72	(0.91–3.27)
Married	223 (59.6)	2.01	(1.15–3.51) *	1.56	(0.86–2.83)	215 (57.5)	1.84	(1.05–3.21) *	1.39	(0.76–2.53)
Widowed	54 (78.3)	4.89	(2.26–10.60) ***	2.43	(1.01–5.86) *	56 (81.2)	5.86	(2.64–12.99) ***	2.94	(1.19–7.29) *
Divorced	25 (42.3)	1.00	Ref.	1.00	Ref.	25 (42.4)	1.00	Ref.	1.00	Ref.
**Professional situation**										
Unemployed	20 (44.4)	1.00	Ref.	1.00	Ref.	23 (51.1)	1.00	Ref.	1.00	Ref.
Professionally active	278 (50.4)	1.27	(0.69–2.34)	1.21	(0.63–2.31)	277 (50.2)	0.96	(0.52–1.77)	0.94	(0.49–1.80)
Pensioner	110 (76.4)	4.04	(2.00–8.18) ***	0.91	(0.37–2.27)	110 (76.4)	3.09	(1.53–6.24) **	0.70	(0.28–1.75)
Student/pupil	73 (47.1)	1.11	(0.57–2.17)	1.57	(0.77–3.22)	70 (45.2)	0.79	(0.40–1.53)	1.07	(0.52–2.19)
**Chronic diseases related to overweight and obesity (e.g., cardiovascular diseases, type II diabetes)**										
Yes	169 (79.7)	4.69	(3.24–6.77) ***	1.81	(1.10–2.97) *	168 (79.2)	4.55	(3.16–6.56) *	1.59	(0.96–2.62)
No	312 (45.6)	1.00	Ref.	1.00	Ref.	312 (45.6)	1.00	Ref.	1.00	Ref.
**Number of chronic diseases**										
0	170 (40.5)	1.00	Ref.	1.00	Ref.	168 (40.0)	1.00	Ref.	1.00	Ref.
1	109 (53.2)	1.67	(1.19–2.34) **	1.41	(0.99–2.00)	106 (51.7)	1.61	(1.15–2.25) **	1.36	(0.95–1.95)
2	67 (61.5)	2.35	(1.52–3.62) ***	1.63	(1.00–2.66) *	69 (63.3)	2.59	(1.67–4.00) ***	1.92	(1.17–3.15) **
≥3	135 (83.3)	7.35	(4.65–11.63) ***	3.03	(1.59–5.76) ***	137 (84.6)	8.22	(5.14–13.16) ***	3.89	(2.01–7.50) ***
**Diseases occurring in the family**										
Diabetes mellitus										
Yes	180 (57.7)	1.28	(0.97–1.69)	1.02	(0.74–1.41)	180 (57.7)	1.29	(0.98–1.70)	1.01	(0.73–1.40)
No	301 (51.5)	1.00	Ref.	1.00	Ref.	300 (51.4)	1.00	Ref.	1.00	Ref.
Coronary artery disease										
Yes	108 (61.7)	1.50	(1.07–2.11) *	1.09	(0.74–1.62)	104 (59.4)	1.34	(0.96–1.88)	0.92	(0.62–1.36)
No	373 (51.8)	1.00	Ref.	1.00	Ref.	376 (52.1)	1.00	Ref.	1.00	Ref.
Neoplastic disease										
Yes	179 (56.6)	1.20	(0.91–1.59)	1.12	(0.82–1.54)	182 (57.6)	1.28	(0.97–1.69)	1.26	(0.91–1.73)
No	302 (59.4)	1.00	Ref.	1.00	Ref.	298 (51.4)	1.00	Ref.	1.00	Ref.
**Subjective assessment of being overweight or obese**										
Yes	209 (71.1)	2.98	(2.21–4.02) ***	1.30	(0.91–1.85) *	211 (71.8)	3.15	(2.33–4.25) ***	1.61	(1.12–2.32) **
No	272 (45.2)	1.00	Ref.	1.00	Ref.	269 (44.7)	1.00	Ref.	1.00	Ref.

* *p* < 0.05, ** *p* < 0.01, *** *p* < 0.001; Fully-adjusted model, including all statistically significant characteristics. Ref-reference; CI-confidence interval.

**Table 4:** Frequency of measurement of body weight and other variables by a GP in primary care patients.

Variables	Body Mass Index BMI									Total *n* = 896		
	<25 kg/m^2^ *n* = 494			≥25–<30 kg/m^2^ *n* = 227			≥30 kg/m^2^ *n* = 175					
	*n* (%)	95% Cl	*p*-Value	*n* (%)	95% Cl	*p*-Value	*n* (%)	95% Cl	*p*-Value	*n* (%)	95% Cl	*p*-Value
**Bodyweight measured on the scale**												
For each routine visit	29 (5.8)	(3.8–7.9)	0.0587	7 (3.1)	(0.8–5.3)	0.0308 *	10 (5.8)	(2.3–9.2)	0.0571	46 (5.1)	(3.7–6.6)	0.0513
Once a year	42 (8.5)	(6.0–11.0)	0.0850	24 (10.6)	(6.6–14.6)	0.1057	20 (11.4)	(6.7–16.1)	0.1143	86(9.6)	(7.7–11.5)	0.0960
If clinically indicated	195 (39.5)	(35.2–43.8)	0.3947	98(43.2)	(36.7–49.6)	0.4317	83(47.4)	(40.0–54.8)	0.4743	376(42.0)	(38.7–45.2)	0.4196
Never	228 (46.2)	(41.8–50.6)	0.4615	98 (43.1)	(36.7–49.6)	0.4317	62 (35.4)	(28.3–42.5)	0.3543	388(43.3)	(40.1–46.5)	0.4330
**Body mass index (BMI)**												
For each routine visit	12 (2.4)	(1.1–3.8)	0.0243 *	3 (1.3)	(−0.2–2.8)	0.0132 *	4 (2.3)	(0.1–4.5)	0.0228 *	19 (2.1)	(1.2–3.1)	0.0212 *
Once a year	21 (4.3)	(2.5–6.0)	0.0425 *	17 (7.5)	(4.1–10.9)	0.0749	27 (15.4)	(10.1–20.8)	0.1543	65(7.3)	(5.6–9.0)	0.0725
If clinically indicated	113(22.9)	(19.2–26.6)	0.2287	64(28.2)	(22.3–34.0)	0.2819	50(28.6)	(21.9–35.3)	0.2857	227(25.3)	(22.5–28.2)	0.2533
Never	348 (70.4)	(66.4–74.5)	0.7044	143(63.0)	(56.7–69.3)	0.6299	94(53.7)	(46.3–61.1)	0.5371	585(65.3)	(62.2–68.4)	0.6529
**Waist**												
For each routine visit	9 (1.8)	(0.6–3.0)	0.0182 *	2 (0.8)	(−0.3–2.1)	0.0088 **	3 (1.7)	(−0.2–3.6)	0.1714	14 (1.5)	(0.8–2.4)	0.0156 *
Once a year	16 (3.2)	(1.7–4.8)	0.0324 *	10 (4.4)	(1.7–7.1)	0.0440 *	8 (4.6)	(1.5–7.7)	0.0457 *	34 (3.8)	(2.5–5.0)	0.0379 *
If clinically indicated	79 (16.0)	(12.8–19.2)	0.1599	55(24.3)	(18.7–29.8)	0.2423	47(26.9)	(20.3–33.4)	0.2686	181(20.2)	(17.6–22.8)	0.2020
Never	390 (79.0)	(75.4–82.5)	0.7895	160(70.5)	(64.5–76.4)	0.7048	117(66.8)	(59.9–73.8)	0.6686	667(74.5)	(71.6–77.3)	0.7444
**Height**												
For each routine visit	37 (7.5)	(5.2–9.8)	0.0749	8 (3.5)	(1.1–5.9)	0.0352 *	11 (6.3)	(2.7–9.9)	0.0628	56 (6.2)	(4.7–7.8)	0.0625
Once a year	43 (8.7)	(6.2–11.2)	0.0870	28 (12.3)	(8.1–16.6)	0.1233	33 (18.9)	(13.1–24.7)	0.1886	104(11.6)	(9.5–13.7)	0.1161
If clinically indicated	188 (38.1)	(33.8–42.3)	0.3806	90 (39.7)	(33.3–46.0)	0.3965	74 (42.3)	(35.0–49.6)	0.4228	352(39.3)	(36.1–42.5)	0.3928
Never	226 (45.7)	(41.4–50.1)	0.4575	101 (44.5)	(38.0–51.0)	0.4449	57 (32.5)	(25.6–39.5)	0.3257	384(42.9)	(39.6–46.1)	0.4286
**Prescribed drug treatment aimed at weight loss**												
Yes	3 (0.6)	(−0.1–1.3)	0.0061 **	3 (1.3)	(−0.2–2.8)	0.0132 *	4 (2.3)	(0.1–4.5)	0.0228 *	10 (1.1)	(0.4–1.8)	0.0111 *
No	364 (73.7)	(69.8–77.6)	0.7368	202 (89.0)	(84.9–93.1)	0.8899	165 (94.3)	(90.8–97.7)	0.9428	731 (81.6)	(79.0–84.1)	0.8158
Not applicable	127 (25.7)	(21.9–29.6)	0.2571	22 (9.7)	(5.8–13.5)	0.0969	6 (3.4)	(0.7–6.1)	0.0343 *	155 (17.3)	(14.8–19.8)	0.1730
**A referral for obesity surgery**												
Yes	1 (0.2)	(−0.2–0.6)	0.0020 **	1 (0.4)	(−0.4–1.3)	0.0044 **	2 (1.1)	(−0.4–2.7)	0.0114 *	4 (0.4)	(0.01–0.9)	0.0045 **
No	345 (69.8)	(65.8–73.9)	0.6984	199 (87.7)	(83.4–91.9)	0.8766	167 (95.5)	(92.3–98.5)	0.9543	711 (79.4)	(76.7–82.0)	0.7935
Not applicable	148 (30.0)	(25.9–34.0)	0.2996	27 (11.9)	(7.7–16.1)	0.1189	6 (3.4)	(0.7–6.1)	0.0343 *	181 (20.2)	(17.6–22.8)	0.2020

* *p* < 0.05, ** *p* < 0.01.

## 4. Discussion

The issue of overweight and obesity is rarely discussed in GPs’ practices. Our cross-sectional study is one of the first in Poland to take up the topic of the nutritional and physical activity counseling provided by family doctors in primary health care during the COVID-19 pandemic. Every third person who joined our study admitted to having received advice on nutrition and physical activity from their GP. Obese patients were advised more often than overweight patients.

However, this counseling was only offered sometimes, not with every routine visit to the GP. Our data do not differ from other studies which show that GPs do not counsel their overweight patients or provide a low level of counseling [21,32,33,34,35,36].

However, the frequency of advice given to obese and overweight people in our study can be considered higher than that reported in other studies, although it cannot be considered satisfactory. In other studies, the frequency of diagnosing overweight in patients by primary care physicians is low [37].

The results of research in Germany [38], France [39], and Hungary [40] showed that most GPs underestimated the prevalence of overweight, considering it as a norm.

A decline in the popularity of counseling related to weight loss and physical activity was also noted in American and German studies [41,42,43]. Obesity is also insufficiently controlled by GPs, as shown by the data from the United Kingdom [24,44]. Less than half of obese patients seeking medical attention have been recommended by their GPs to lose weight or take up physical activity [45,46,47].

In some studies, the advice on exercise and nutrition was fairly general, without giving detailed strategies [48,49]. Similarly, in our study, the advice given to patients was mainly related to general tips on changing diet, exercise, or weight loss. However, our study found that detailed advice on diet/nutrition and exercise was also given. GPs recommended specific nutrients and foods or eating behaviors, as in other studies [50,51,52].

A doctor’s advice can help patients achieve positive behavioral changes and promote weight loss efforts [53].

Motivating patients to take responsibility for their health should be the main objective of GP consultation in the treatment of obesity [54]. A lack of patient motivation is the main barrier to the effective management of the problem in primary care [54]. Motivational interviewing (MI) can improve weight attitudes and behavior and thus lead to improved weight loss [55,56]. There was a correlation between the advice received and the patient’s attempt to lose weight [57].

Research shows that MI increases weight loss in overweight and obese people as compared to those who were not provided with such an intervention [55,56].

The low percentage of advice on diet and exercise reported by overweight patients suggests that more lifestyle advice should be offered in primary care [57]. It is important to adapt counseling to the individual needs of each patient [58].

The results of our study show that discussions about nutrition and physical activity were more often undertaken by GPs than by patients. Similarly, in other studies, GPs were more likely to initiate most weight discussions than patients [22], and they also measured body weight [59].

Patients prefer to receive weight management support from GPs rather than other health care professionals [23].

However, some patients have little confidence in their GP regarding obesity management and do not feel sufficiently supported in their weight control [60,61]. Research shows that some patients consider it to be their own (not their doctor’s) responsibility to control their weight [62].

Our study reported a low percentage of patient’s body weight, height, and waist measurements performed by GPs. Australian and German studies also showed that routine body weight measurements were rare [63]. Such measurements should be an integral part of the physical examination for overweight and obesity and should be performed at least annually [5]. It is obligatory during the first visit to the family doctor to have the patient’s weight, height, and waist circumference measured.

Our study found that the likelihood of being provided with counseling was related to BMI. GPs were more likely to give advice when the BMI was high, as in other studies [57].

The obesity management guidelines of the National Institute for Health and Care Excellence (NICE) focus on the concept of patient-centered care, advising that the choice of weight management interventions should be discussed and agreed upon with each individual patient [64]. Polish and foreign guidelines recommend a routine identification of obesity in primary health care, using BMI (body mass index) as a practical assessment of obesity in adults [25,64,65].

Our study found that as age and comorbidities increased, so did the percentage of people advised by GPs. Similarly, in other studies, physicians were more likely to provide weight management counseling to patients with comorbidities, including those related to obesity, than to overweight and obese patients without risk factors [66,67].

Overweight and obesity are rarely treated as a single disease, most often being managed in combination with other diseases such as cardiovascular disease [54].

Our study found that GPs’ advice for overweight patients depended on the patient’s socio-demographic characteristics. The subjects aged 30–39 and 40–49 years and the widowed patients were more likely to receive advice on physical activity. In the Swedish study, men, younger, and better-educated patients were consulted more often by their family doctor [21]. Other authors have shown that a higher percentage of women and older people received counseling [36].

The most common barrier related to the poor counseling mentioned by doctors and patients is the time limit of the medical appointment for each patient [47,63]. A successful intervention and follow-up of the patient require time. Due to the short visiting times, comprehensive lifestyle recommendations may not be practicable.

Some GPs argue that primary care is not an appropriate resource for intervention and that patients are responsible for their obesity [68,69]. Many doctors indicate a lack of motivation to change patients’ behavior and a lack of self-efficacy [45,54,68,70,71].

It has been shown that coordination between primary and specialist health care for obese patients is poorly developed [72]. Advice that suggests dietary modification and increased physical activity is given more often than referrals [73]. This was also confirmed by our study, in which a small percentage of overweight and obese respondents were referred by their family doctor to another health specialist.

The current literature shows that GPs are essential for engaging patients in interventions aimed at reducing their overweight and obesity [63].

The strengths and limitations of the study are as follows. This is the latest study on the impact of a family doctor on the health behaviors of overweight and obese patients in Poland conducted during the COVID-19 pandemic. There are not many studies on this issue in Poland, and it is the first such study to provide information on the scale of counseling provided during the COVID-19 pandemic.

This study describes the urban population which ensures the generalization of the results for other urban areas and other populations. The advantage of this study is the examination of many determinants that may influence the counseling of family doctors among the studied population.

This study also has limitations. It was cross-sectional and carried out over a single time point, which makes it impossible to observe changes over longer periods. To obtain health benefits, a several-month intervention is insufficient; instead, an intervention must be carried out over a long-term.

This study was also limited by the study period during the COVID-19 pandemic. It was associated with difficult access to patients, and not all patients visited their family doctor in this period.

Nutrition and physical activity counseling by GPs were assessed using patient self-reported questionnaire data, which may be associated with recall bias.

The questionnaire did not include a question on the reason for which the patient came to the doctor; however, regardless of the cause, the family doctor should always notice overweight and obesity and encourage the patient to start treatment.

The lack of association in multivariate analyses may be caused by the small sample size. Moreover, in the current study, we have evaluated the patient-related variables (socio-demographic or health related factors). However, the other variables, including doctor-related ones, which were not evaluated in the present study, may be more important correlates of counseling provided by primary care physicians.

Additionally, the results of the research may be important for the development of health programs aimed at reducing overweight and obesity in Poland and other countries.

Our study was anonymous, and thus the respondents could not be linked to their GPs in any way.

## 5. Conclusions

The study identified correlates of higher counseling for overweight and obesity-related chronic diseases. The percentage of advice in Poland on diet and physical activity from primary care physicians reported by overweight and obese patients remains low.

The study found a higher proportion of advice on diet and physical activity provided by primary care physicians to overweight and obese patients than in other studies; however, still not all patients receive the necessary counseling.

Primary care physicians should advise all patients not to be overweight and obese, not only those already affected by the problem. The study shows that primary care physicians focus on the treatment of overweight and obesity, but not its prevention. Early prophylaxis is important as it ensures more benefits for the patient than a modification of the treatment. Family doctors should raise awareness and shape the pro-health attitudes of their patients.

Counseling appears to have a significant impact on changing the health behaviors related to weight control among patients, which is a priority for improving the health of the population. It is important to study the work of doctors in the field of overweight and obesity counseling. There is a need for additional efforts to increase the frequency of the abovementioned advice in primary care. Determining the importance of primary health care counseling by the National Health Fund will be helpful in this respect. When planning prevention programs, healthcare managers in Lodz and other cities in Poland should consider counseling as a part of the regular primary healthcare services.

## Figures and Tables

**Table 1:** Characteristics of the studied population.

Characteristics	Total *n* = 896	%
**Gender**		
Males	231	25.8
Females	665	74.2
**Age (years)**		
<30	256	28.6
30–39	123	13.7
40–49	215	24.0
50–59	105	11.7
60+	197	22.0
**Education**		
Primary	26	2.9
Medium/Secondary	510	56.9
Post-secondary vocational	74	8.3
Higher	286	31.9
**Body mass index BMI**		
<25 kg/m ^2^	494	55.1
≥25–<30 kg/m ^2^	227	25.4
≥30 kg/m ^2^	175	19.5
**Marital status**		
Single	394	44.0
Married	374	41.7
Widowed	69	7.7
Divorced	59	6.6
**Professional situation**		
Unemployed	45	5.0
Professionally active	552	61.6
Pensioner	144	16.1
Student/pupil	155	17.3
**Chronic diseases related to overweight and obesity (e.g., cardiovascular diseases, type II diabetes)**		
Yes	212	23.7
No	684	76.3
**Number of chronic diseases**		
0	420	46.9
1	205	22.9
2	109	12.2
≥3	162	18.0
**Diseases occurring in the family**		
Diabetes mellitus	312	34.8
Coronary artery disease	175	19.5
Neoplastic disease	316	35.3
Other	47	5.2
**Subjective assessment of being overweight or obese**		
Yes	294	32.8
No	602	67.2

## Data Availability

The datasets used and/or analyzed during the current study are available from the corresponding author upon reasonable request.

## Abbreviations

GP	General Practitioner
WHO	World Health Organization
BMI	Body Mass Index
MI	Motivational Interviewing
NICE	The National Institute for Health and Care Excellence

## References

[1] Kivimaki M., Kuosma E., Ferrie J.E., Luukkonen R., Nyberg S.T., Alfredsson L., Batty G.D., Brunner E.J., Fransson E., Goldberg M. (2017). Overweight, obesity, and risk of cardiometabolic multimorbidity: A pooled analysis of individual-level data for 120 813 adults from 16 cohort studies from the USA and Europe. Lancet Public Health.

[2] Jovic D., Marinkovic J., Vukovic D. (2016). Association between body mass index and prevalence of multimorbidity: A cross-sectional study. Public Health.

[3] Fruhbeck G., Toplak H., Woodward E. (2013). Obesity: The gateway to ill health—An EASO position statement on rising public health, the clinical and scientific challenge in Europe. Obes. Facts.

[4] Durrer Schutz D., ·Busetto L., Dicker D., Farpour-Lambert N., Pryke R., Toplak H., Widmer D., Yumuk V., Schutz Y. (2019). European Practical and Patient-Centred Guidelines for Adult Obesity Management in Primary Care. Obes. Facts.

[5] Olszanecka-Glinianowicz M., Godycki-Ćwirko M., Lukas W., Mastalerz-Migas A., Tomasik T., Tomiak E., Windak A. (2017). Zasady postępowania w nadwadze i otyłości w praktyce lekarza rodzinnego Wytyczne Kolegium Lekarzy Rodzinnych w Polsce, Polskiego Towarzystwa Medycyny Rodzinnej oraz Polskiego Towarzystwa Badań nad Otyłością. Lek. Rodz.-Wyd. Spec..

[6] (2021). WHO Discussion Paper. Draft Recommendations for the Prevention and Management of Obesity over the Life Course, Including Potential Targets. https://cdn.who.int/media/docs/default-source/obesity/who-discussion-paper-on-obesity---final190821.pdf.

[7] Otyłość w UE, Polsce i na świecie. Choroba XXI Wieku. https://demagog.org.pl/analizy_i_raporty/otylosc-w-ue-polsce-i-na-swiecie-choroba-xxi-wieku/?cn-reloaded=1.

[8] Eurostat Over Half of Adults in the EU Are Overweight. https://ec.europa.eu/eurostat/web/products-eurostat-news/-/ddn-20210721-2.

[9] Wojtyniak B., Goryński P. (2020). Sytuacja Zdrowotna ludności Polski i jej Uwarunkowania 2020.

[10] World Health Organization (2022). World Obesity Day 2022- Accelerating Action to Stop Obesity. https://www.who.int/news/item/04-03-2022-world-obesity-day-2022-accelerating-action-to-stop-obesity.

[11] Ostrowska L., Bogdański P., Mamcarz A. (2021). Otyłość i jej Powikłania. Praktyczne Zalecenia Diagnostyczne i Terapeutyczne.

[12] Salas-Salvado J., Diaz-Lopez A., Ruiz-Canela M., Basora J., Fito M., Corella D., Serra-Majem L., Wärnberg J., Romaguera D., Estruch R. (2019). Effect of a Lifestyle Intervention Program with Energy-Restricted Mediterranean Diet and Exercise on Weight Loss and Cardiovascular Risk Factors: One-Year Results of the PREDIMED-Plus Trial. Diabetes Care.

[13] Ma C., Avenell A., Bolland M., Hudson J., Stewart F., Robertson C., Sharma P., Fraser C., MacLennan G. (2017). Effects of weight loss interventions for adults who are obese on mortality, cardiovascular disease, and cancer: Systematic review and meta-analysis. BMJ.

[14] Patnode C.D., Evans C.V., Senger C.A., Redmond N., Lin J.S. (2017). Behavioral Counseling to Promote a Healthful Diet and Physical Activity for Cardiovascular Disease Prevention in Adults Without Known Cardiovascular Disease Risk Factors: Updated Systematic Review for the U.S. Preventive Services Task Force. JAMA.

[15] Ustawa z Dnia 27.10.2017r. o Podstawowej Opiece Zdrowotnej (Dz.U. z 2017, poz. 2217). https://sap.sejm.gov.pl/isap.nsf/download.xsp/WDU20170002217/U/D20172217Lj.pdf.

[16] Burns P., Jones S.C., Iverson D.C., Caputi P. (2013). Where do older Australians receive their health information? Health information sources and their perceived reliability. J. Nurs. Educ. Pract..

[17] Pollak K.I., Coffman C.J., Alexander S.C., Østbye T., Lyna P., Tulsky J.A., Bilheimer A., Dolor R.J., Lin P.H., Bodner M.E. (2014). Weight’s up? Predictors of weight-related communication during primary care visits with overweight adolescents. Patient Educ. Couns..

[18] Lannoy L., Cowan T., Fernandez A., Ross R. (2021). Physical activity, diet, and weight loss in patients recruited from primary care settings: An update on obesity management interventions. Obes. Sci. Pract..

[19] Rodondi N., Humair J.P., Ghali W.A., Ruffieux C., Stoianov R., Seematter-Bagnoud L., Stalder H., Pecoud A., Cornuz J. (2006). Counselling overweight and obese patients in primary care: A prospective cohort study. Eur. J. Cardiovasc. Prev. Rehabil..

[20] Fruhbeck G., Toplak H., Woodward E., Halford J.C., Yumuk V. (2014). Need for a paradigm shift in adult overweight and obesity management—An EASO position statement on pressing public health, clinical and scientific challenge in Europe. Obes. Facts.

[21] Brobeck E., Bergh H., Odencrants S., Hildingh C. (2015). Lifestyle advice, and lifestyle change: To what degree does lifestyle advice of healthcare professionals reach the population, focusing on gender, age, and education?. Scand. J. Caring Sci..

[22] Gray L., Stubbe M., Macdonald L., Tester R., Hilder J., Dowell A.C. (2018). A taboo topic? How General Practitioners talk about overweight and obesity in New Zealand. J. Prim. Health Care.

[23] Jansen S., Desbrow B., Ball L. (2015). Obesity management by general practitioners: The unavoidable necessity. Aust. J. Prim. Health.

[24] McLaughlin J.C., Hamilton K., Kipping R. (2017). Epidemiology of adult overweight recording and management by UK GPs: A systematic review. Br. J. Gen. Pract..

[25] Olszanecka-Glinianowicz M., Dudek D., Filipiak K.J., Krzystanek M., Markuszewski L., Ruchała M., Tomiak E. (2020). Wytyczne. Leczenie nadwagi i otyłości w czasie i po pandemii. Nie czekajmy na rozwój powikłań—Nowe wytyczne dla lekarzy. Nutr. Obes. Metab. Surg..

[26] Rajca A., Wojciechowska A., Śmigielski W., Drygas W., Piwońska A., Pająk A., Tykarski A., Kozakiewicz K., Kwaśniewska M., Zdrojewski T. (2021). Increase in the prevalence of metabolic syndrome in Poland: Comparison of the results of the WOBASZ (2003–2005) and WOBASZ II (2013–2014) studies. Pol. Arch. Intern. Med..

[27] (2020). Rocznik Statystyczny woj. Łódzkiego 2020. https://lodz.stat.gov.pl/publikacje-i-foldery/roczniki-statystyczne/rocznik-statystyczny-wojewodztwa-lodzkiego-2020,6,22.html#.

[28] OECD/European Union (2020). Health at a Glance: Europe 2020. State of Health in the EU Cycle.

[29] Kalucka S., Kaleta D., Makowiec- Dąbrowska T. (2019). Prevalence of Dietary Behavior and Determinants of Quality of Diet among Beneficiaries of Government Welfare Assistance in Poland. Int. J. Environ. Res. Public Health.

[30] Kaleta D., Kalucka S., Szatko F. (2017). Prevalence and correlates of physical inactivity during leisure-time and commuting among beneficiaries of Government Welfare Assistance in Poland. Int. J. Environ. Res. Public Health.

[31] Body Mass Index. http://www.euro.who.int/en/health-topics/disease-prevention/nutrition/a-healthy-lifestyle/body-mass-index-bmi.

[32] Van Dillen S.M., van Binsbergen J.J., Koelen M.A., Hiddink G.J. (2013). Nutrition and physical activity guidance practices in general practice: A critical review. Patient Educ. Couns..

[33] Kriaucioniene V., Petkeviciene J., Raskiliene A. (2019). Nutrition and physical activity counseling by general practitioners in Lithuania, 2000–2014. BMC Fam. Pract..

[34] Booth A.O., Nowson C.A. (2010). Patient recall of receiving lifestyle advice for overweight and hypertension from their general practitioner. BMC Fam. Pract..

[35] Pickett-Blakely O., Bleich S., Cooper L.A. (2011). Patient-physician gender concordance and weight-related counseling of obese patients. Am. J. Prev. Med..

[36] Booth H.P., Prevost A.T., Gulliford M.C. (2015). Access to weight reduction interventions for overweight and obese patients in UK primary care: Population-based cohort study. BMJ Open.

[37] Bramlage P., Wittchen H.U., Pittrow D., Kirch W., Krause P., Lehnert H., Unger T., Hoflera M., Küpper B., Dahm S. (2004). Recognition and management of overweight and obesity in primary care in Germany. Int. J. Obes. Relat. Metab. Disord..

[38] Schwenke M., Luppa M., Pabst A., Welzel F.D., Löbner M., Luck-Sikorski C., Kersting A., Blüher M., Riedel-Heller S.G. (2020). Attitudes and treatment practice of general practitioners towards patients with obesity in primary care. BMC Fam. Pract..

[39] Bocquier A., Verger P., Basdevant A., Andreotti G., Baretge J., Villani P., Paraponaris A. (2005). Overweight, and obesity: Knowledge, attitudes, and practices of general practitioners in France. Obes. Res..

[40] Rurik I., Torzsa P., Ilyés I., Szigethy E., Halmy E., Iski G., Kolozsvári L.R., Mester L., Móczár C., Rinfel J. (2013). Primary care obesity Management in Hungary: Evaluation of the knowledge, Practice, and Attitudes of Family Physicians. BMC Fam. Pract..

[41] Tomiyama A.J., Finch L.E., Belsky A.C.I., Buss J., Finley C., Schwartz M.B., Daubenmier J. (2015). Weight bias in 2001 versus 2013: Contradictory attitudes among obesity researchers and health professionals. Obesity.

[42] Schwartz M.B., Chambliss H.O., Brownell K.D., Blair S.N., Billington C. (2003). Weight bias among health professionals specializing in obesity. Obes. Res..

[43] Puhl R.M., Latner J.D., O’Brien K., Luedicke J., Danielsdottir S., Forhan M. (2015). A multinational examination of weight bias: Predictors of anti-fat attitudes across four countries. Int. J. Obes..

[44] Sturgiss E.A., Elmitt N., Haesler E., van Weel C., Douglas K.A. (2018). Role of the family doctor in the management of adults with obesity: A scoping review. BMJ Open.

[45] Foster G.D., Wadden T.A., Makris A.P., Davidson D., Sanderson R.S., Allison D.B., Kessler A. (2003). Primary care physicians’ attitudes about obesity and its treatment. Obes. Res..

[46] Lobelo F., Duperly J., Frank E. (2009). Physical activity habits of doctors and medical students influence their counseling practices. Br. J. Sports Med..

[47] Wadden T.A., Volger S., Sarwer D.B., Vetter M.L., Tsai A.G., Berkowitz R.I., Kumanyika S., Schmitz K.H., Diewald L.K., Barg R. (2011). A two-year randomized trial of obesity treatment in primary care practice. N. Engl. J. Med..

[48] Curtis S.M., Willis M.S. (2016). Are you eating healthy? Nutrition discourse in Midwestern clinics for the underserved. Patient Educ. Couns..

[49] Heintze C., Metz U., Hahn D., Niewohner J., Schwantes U., Wiesner J., Broun V. (2010). Counseling overweight in primary care: An analysis of patient-physician encounters. Patient Educ. Couns..

[50] Stanford F.C., Durkin M.W., Stallworth J.R., Powell C.K., Poston M.B., Blair S.N. (2014). Factors that influence physicians and medical students’ confidence in counseling patients about physical activity. J. Prim. Prev..

[51] Nicklas J.M., Huskey K.W., Davis R.B., Wee C.C. (2012). Successful weight loss among obese U.S. adults. Am. J. Prev. Med..

[52] Santos I., Sniehotta F.F., Marques M.M., Carraca E.V., Teixeira P.J. (2017). Prevalence of personal weight control attempts in adults: A systematic review and meta-analysis. Obes. Rev..

[53] Rueda-Clausen C.F., Benterud E., Bond T., Olszowka R., Vallis M.T., Sharma A.M. (2014). Effect of implementing the 5As of obesity management framework on provider-patient interactions in primary care. Clin. Obes..

[54] Sonntag U., Brink A., Renneberg B., Braun V., Heintze C. (2012). GPs’ attitudes, objectives and barriers in counseling for obesity—A qualitative study. Eur. J. Gen. Pract..

[55] Armstrong M.J., Mottershead T.A., Ronksley P.E., Sigal R.J., Campbell T.S., Hemmelgarn B.R. (2011). Motivational interviewing to improve weight loss in overweight and/or obese patients: A systematic review and meta-analysis of randomized controlled trials. Obes. Rev..

[56] Cox M.E., Yancy W.S., Coffman C.J., Ostbye T., Tulsky J.A., Alexander S.C., Brouwer R.J., Dolor R.J., Pollak K.I. (2011). Effects of counseling techniques on patients’ weight-related attitudes and behaviors in a primary care clinic. Patient Educ. Couns..

[57] Klumbiene J., Petkeviciene J., Vaisvalavicius V., Miseviciene I. (2006). Advising overweight persons about diet and physical activity in primary health care: Lithuanian health behavior monitoring study. BMC Public Health.

[58] McGowan B.M. (2016). A Practical Guide to Engaging Individuals with Obesity. Obes. Facts.

[59] Prod’homme L., Riglet C., Godart N., Huas C. (2019). [Climb on the scale! Weight in consultations and adult patient’s experiences: Exploratory study in general practice]. Sante Publique.

[60] Malterud K., Ulriksen K. (2010). Obesity in general practice. Scand. J. Prim. Health Care.

[61] Ely A.C., Befort C., Banitt A., Gibson C., Sullivan D. (2009). A qualitative assessment of weight control among rural Kansas women. J. Nutr. Educ. Behav..

[62] Heintze C., Sonntag U., Brinck A., Huppertz M., Niewöhner J., Wiesner J., Braun V. (2012). A qualitative study on patients’ and physicians’ visions for the future management of overweight or obesity. Fam. Pract..

[63] Glenister K.M., Malatzky C.A., Wright J. (2017). Barriers to effective conversations regarding overweight and obesity in regional Victoria. Aust. Fam. Physician.

[64] National Institute for Health and Care Excellence (2014). Obesity: Identification, Assessment, and Management of Overweight and Obesity in Children, Young People, and Adults. www.nice.org.uk/guidance/cg189.

[65] National Institute for Health and Care Excellence (2014). Weight Management: Lifestyle Services for Overweight or Obese Adults. PH53.

[66] Kilpatrick M., Nelson M., Palmer A., Jose K., Venn A. (2018). Who discusses reaching a healthy weight with a general practitioner? Findings from the 2014–15 Australian National Health Survey. Obes. Res. Clin. Pract..

[67] Simkin-Silverman L.R., Gleason K.A., King W.C., Weissfeld L.A., Buhari A., Boraz M.A., Wing R.R. (2005). Predictors of weight control advice in primary care practices: Patient health and psychosocial characteristics. Prev. Med..

[68] Epstein L., Ogden J. (2005). A qualitative study of GPs’ views of treating obesity. Br. J. Gen. Pract..

[69] Henderson E. (2015). Obesity in primary care: A qualitative synthesis of patient and practitioner perspectives on roles and responsibilities. Br. J. Gen. Pract..

[70] Salinas G.D., Glauser T.A., Williamson J.C., Rao G., Abdolrasulnia M. (2011). Primary care physician attitudes and practice patterns in the management of obese adults: Results from a national survey. Postgrad. Med..

[71] Thuan J.F., Avignon A. (2005). Obesity management: Attitudes and practices of French general practitioners in a region of France. Int. J. Obes. Relat. Metab. Disord..

[72] Osmundsen T.C., Dahl U., Kulseng B. (2019). Enhancing knowledge and coordination in obesity treatment: A case study of an innovative educational program. BMC Health Serv. Res..

[73] Critchlow N., Rosenberg G., Rumgay H., Petty R., Vohra J. (2020). Weight assessment and the provision of weight management advice in primary care: A cross-sectional survey of self-reported practice among general practitioners and practice nurses in the United Kingdom. BMC Fam. Pract..

